# Dual-task, single challenge: functionality as an early marker of cognitive decline

**DOI:** 10.1590/1980-5764-DN-2025-0349

**Published:** 2026-08-14

**Authors:** Júlia Silva de Almeida, Isabelle Louise da Silva Rosendo, Jessica dos Santos Sacramento Plácido, Felipe de Oliveira Silva, José Vinícius Alves Ferreira, Luiz Felipe da Silva Figueiredo, Andrea Camaz Deslandes, Daniella Cristina de Assis Pinto Gomes

**Affiliations:** 1 Universidade Federal do Rio de Janeiro, Instituto de Psiquiatria, Rio de Janeiro, RJ, Brazil.; 2 Universidade Federal do Rio de Janeiro, Instituto de Psiquiatria, Programa de Pós-Graduação em Psiquiatria e Saúde Mental, Rio de Janeiro, RJ, Brazil.

**Keywords:** Aged, Functional Status, Multitasking Behavior, Biomarkers, Cognition, Idoso, Estado Funcional, Comportamento Multitarefa, Biomarcadores, Cognição

## Abstract

Early detection of possible cognitive decline (PwCD) is crucial for preserving function and independence in aging.

**Objective:**

To examine whether dual-task and functional assessment tools can help detect cognitive impairment in older adults without prior diagnosis of neurocognitive disorders.

**Methods:**

A cross-sectional study was conducted with healthy older adults (≥60 years). The following exclusion criteria were adopted: neurological diseases with motor deficits, severe functional limitations, mobility aid use, and inability to walk six meters unaided. Assessments included the Mini-Mental State Examination (MMSE), Montreal Cognitive Assessment (MoCA), Clock Drawing Test (CDT), Verbal Fluency (VF), Pfeffer Functional Activities Questionnaire, Timed Up and Go (TUG), Sit-to-Stand Test (STS), Berg Balance Scale (BBS), Step Test, and dual-task TUG (TUGdt) using animal naming (TUGdt-NA) and serial subtraction (TUGdt-S7). Statistical analyses included t-tests, Mann-Whitney U, ꭓ^2^ tests, and logistic regression (p≤0.05).

**Results:**

Among 144 participants, 89 were classified as healthy and 55 as PwCD. Most were female (90.9%), black (57.7%), with an average of 11.04 years of education. Groups did not differ in age, gender, or comorbidities. The PwCD group had lower education (p=0.01), more black individuals (p=0.03), and scored significantly worse on all cognitive tests (p<0.0001) and TUGdt (NA: p=0.040; S7: time p=0.027; accuracy p<0.001). No differences were found in STS, Step Test, BBS, or total Pfeffer scores; however, the PwCD group showed greater instrumental activities of daily living (IADL) impairment.

**Conclusion:**

The ability to perform dual tasks appears to be associated with functional capacity.

## RESUMO

A detecção precoce do possível declínio cognitivo (PwCD) é fundamental para preservar a funcionalidade e a independência no envelhecimento.

**Objetivo:**

Investigar se ferramentas de avaliação funcional e dupla tarefa podem auxiliar na identificação do declínio cognitivo em idosos sem diagnóstico prévio de transtornos neurocognitivos.

**Métodos:**

Trata-se de estudo transversal com idosos saudáveis (≥60 anos). Foram adotados como critérios de exclusão: doenças neurológicas com déficits motores, incapacidade funcional grave, uso de auxílios de locomoção e incapacidade de caminhar seis metros sem assistência. As avaliações incluíram Mini-Exame do Estado Mental (MEEM), Montreal Cognitive Assessment (MoCA), Teste de Desenho do Relógio (TDR), Fluência Verbal (FV), Questionário de Atividades Funcionais de Pfeffer, Timed Up and Go (TUG), Teste de Sentar e Levantar (TSL), Escala de Equilíbrio de Berg (EEB), Step Test e TUG com dupla tarefa (TUGdt) nomeando animais (TUGdt-NA) e subtraindo em série (TUGdt-S7). As análises estatísticas envolveram testes t, Mann-Whitney U, ꭓ^2^ e regressão logística (p≤0,05).

**Resultados:**

Entre 144 participantes, 89 foram considerados saudáveis e 55 com PwCD. A maioria era de mulheres (90,9%), negros (57,7%) e com escolaridade média de 11,04 anos. Os grupos não diferiram em idade, gênero ou comorbidades. O grupo PwCD apresentou menor escolaridade (p=0,01), maior proporção de negros (p=0,03), além de pontuações inferiores em todos os testes cognitivos (p<0,0001) e no TUGdt (NA: p=0,040; S7-tempo: p=0,027; acurácia: p<0,001). Não foram observadas diferenças no TSL, Step Test, EEB ou pontuação total de Pfeffer; porém, o grupo PwCD demonstrou maior comprometimento nas atividades instrumentais de vida diária (AIVDs).

**Conclusão:**

O desempenho em dupla tarefa pode estar associado à capacidade funcional do indivíduo.

## INTRODUCTION

Currently, approximately 19.7% of older adults worldwide are estimated to have some degree of cognitive impairment, and this prevalence is expected to double in the coming years^1^. Early identification of preclinical stages is essential for implementing strategies aimed at preserving functional capacity and independence during aging, as well as reducing the incidence of dementia^2^. Nevertheless, up to 50% of cases of mild cognitive impairment (MCI) remain undiagnosed^3,4^, largely because early symptoms are often misattributed to normal aging. MCI is characterized by a decline in one or more cognitive domains beyond what is expected for age and educational level, while functional independence is preserved, often through compensatory strategies^5^. Annual conversion rates from MCI to dementia range from 10 to 15%^6,7^; however, a proportion of cases may remain stable or even revert to normal cognition, with reported rates of 29 to 55% and 4 to 15%, respectively^8^.

In clinical practice, cognitive screening for MCI commonly relies on instruments such as the Montreal Cognitive Assessment (MoCA) and the Mini-Mental State Examination (MMSE)^9,10,11^. However, evidence suggests that these tools may have limited sensitivity for detecting early cognitive decline, particularly due to the influence of educational level and cultural variability^11^. In addition, their administration requires trained professionals, and they are often applied only after cognitive changes become clinically evident or following patient or family concerns.

Recent studies have highlighted the potential of motor-based assessments for identifying MCI, as well as depressive symptoms^12,13,14^. Montero-Odasso et al.^12^ demonstrated that, beyond cognitive symptoms, MCI is associated with a distinct “motor signature” detectable through gait speed assessments. Similarly, Oliveira Silva et al.^13^ reported significant differences in performance time during single- and dual-task (DT) gait tests between healthy older adults and those with MCI. According to the subcommittee of the Canadian Consensus Conference on the Diagnosis and Treatment of Dementia^15^, gait speed may serve as an early biomarker of dementia, and its monitoring — particularly under DT conditions — may help identify older adults at increased risk of cognitive decline. Grounded in capacity-sharing, bottleneck, and multiple-resource theories, tasks requiring simultaneous cognitive and motor demands tend to be more sensitive than purely motor tasks due to increased mental workload and performance costs. Supporting this notion, a recent meta-analysis demonstrated that different stages of cognitive impairment and dementia can be distinguished using the Timed Up and Go (TUG) test, reinforcing the value of incorporating functional assessments into cognitive screening batteries, especially given their minimal influence of educational level^13^.

Additionally, Oliveira Silva et al.^16^ identified an association between motor-cognitive decline and partial dependence in instrumental activities of daily living (IADLs) among older adults with dementia. Participants were classified according to levels of partial IADL dependence, revealing a direct relationship between cognitive impairment and functional limitations. Notably, individuals with dementia exhibited greater impairment in motor-cognitive performance than in motor or cognitive tasks assessed separately. These findings suggest that DT performance may serve as a useful screening tool for identifying functional capacity in older adults with dementia. Therefore, considering the relevance of early screening for cognitive and functional decline, along with the simplicity and low cost of DT and functional assessments, the present study aimed to explore the applicability of these tools in older adults without a prior diagnosis of cognitive impairment, with the goal of identifying individuals at risk.

## METHODS

This cross-sectional, descriptive, and observational study used data from the Acompanhar Project, a cohort study approved by the Ethics Committee of the Institute of Psychiatry of the Federal University of Rio de Janeiro (CAAE: 82714118.4.0000.5263). All participants provided informed and voluntary consent prior to enrollment. The sample consisted of community-dwelling older adults of both sexes aged 60 years or older who participated in programs promoting healthy aging through physical activities and social and cognitive enrichment. Individuals with severe hearing or visual impairments, neurological diseases associated with motor deficits, severe functional disability (New York Heart Association classes III and IV), use of mobility aids, or inability to walk more than six meters independently were excluded.

Participants were recruited from population lists provided by the Social Service of Commerce (SESC) and community centers and were invited to participate in the study. During the first visit, participants signed the informed consent form and underwent interviews, medical history assessments, and neuropsychological evaluations. Eligible individuals subsequently completed motor and functional assessments conducted at SESC facilities (in the Madureira and Copacabana neighborhoods of Rio de Janeiro) and at the Mangueira Olympic Village, also located in Rio de Janeiro.

For cognitive assessment, the following instruments were used: the MMSE, with education-adjusted cutoff scores established for the exclusion of suspected cognitive impairment (13 points for illiterate individuals, 18 points for those with 1–7 years of schooling, and 26 points for individuals with 8 or more years of education)^17^; the MoCA, used to assess global cognition, with a cutoff score of 26 points regardless of educational level^10^; the Clock Drawing Test (CDT), which evaluates visuospatial and executive functions by asking participants to draw a clock face with numbers and to position the hands to indicate the time requested by the examiner. The test is scored from 0 to 3, with scores ≥2 indicating adequate performance^18^; and the Verbal Fluency Test (VF), used to assess semantic memory and executive function, with cutoff values of 9 animals for individuals with fewer than 9 years of schooling and 12 animals for those with 9 or more years of education^19^.

Motor performance was evaluated using four tests: the TUG test^20^ to assess mobility and dynamic balance; the Sit-to-Stand Test (STS)^21^ to evaluate lower limb strength; the Berg Balance Scale (BBS)^22^ to measure functional balance; and the Step Test^21^ to assess aerobic capacity and cardiovascular endurance. Functional ability was assessed using the Pfeffer Functional Activities Questionnaire^23^, a 10-item instrument that evaluates independence in IADLs. The questionnaire was administered to the participants, and functional impairment was defined as a score of 3 or higher.

DT performance was assessed using the TUG combined with cognitive tasks (TUGdt). In the TUGdt with animal naming (TUGdt-NA)^24^, participants named animals while performing the test, with analysis of execution time, gait speed, and number of animals recalled. In the TUGdt with serial subtractions (TUGdt-S7)^25,26^, participants performed serial subtractions while walking, and correct and incorrect responses were recorded. Dual-task cost (DTC) was calculated as the percentage difference between TUGdt and single-task TUG execution times using the formula: [(TUGdt - TUG)/TUG] × 100^27^.

Participants were objectively classified into two groups, possible cognitive decline (PwCD) and healthy, based on cognitive test performance. Individuals scoring below the established cutoff on at least one of the instruments, MMSE, MoCA, or VF, were considered at risk for cognitive decline and assigned to the PwCD group. Participants with a prior diagnosis of mild cognitive impairment were excluded from the sample.

Data normality and homoscedasticity were assessed using the Kolmogorov-Smirnov and Levene tests, respectively. Between-group comparisons (PwCD vs. healthy) were performed using independent t-tests for parametric data and the Mann–Whitney U test for nonparametric data. Categorical variables were analyzed using the ꭓ^2^ test, and logistic regression was conducted to examine associations between DT performance and functional ability. Statistical significance was set at p≤0.05. Missing data were excluded from the analyses. All statistical procedures were performed using Statistical Package for the Social Sciences (SPSS^®^), version 26.0 (IBM Corporation, New York, USA).

## RESULTS

Initially, 182 individuals were recruited; 39 were excluded due to dementia (n=3), Parkinson’s disease (n=1), or mood and anxiety disorders (n=35). Thus, 144 older adults were included and divided into two groups: Healthy (n=89) and PwCD (n=55). The sample was predominantly female (90.9%), self-identified as black (57.7%), with a median of 11 years of education. When stratified by possible cognitive decline, no differences were observed between groups in age, sex, or number of comorbidities; however, the PwCD group had lower educational attainment and a higher proportion of self-identified black individuals (Table 1).

**Table 1:** Descriptive analysis, comparative analysis of cognitive, motor and functionality variables between groups.

	PwCD (n=55)	Healthy (n=89)	ꭓ^2^	U/T	p-value
Age (years)	71.40±6.297	72.15±5.544		2184.5	0.270
Schooling (years)	9.83±4.195	11.77±4.372		1669.0	0.010
Comorbidity (number)	1.85±1.071	2.14±1.456		2127.5	0.280
Sex (%)					
Female	35.4	64.6	3.4		0.640
Male	61.5	38.5			
Race (%)					
Black	64.33%	42.96%	4.2		0.030
White	35.67%	57.04%			
MMSE (score)	25.04±2.848	27.74±1.410		884.5	<0.0001
MoCA (score)	18.16±3.660	23.63±3.149		637.5	<0.0001
CDT (score)	1.29±0.975	2.67±0.471		661.5	<0.0001
VF (score)	13.78±4.241	17.87±4.320		1165.0	0.0001
STS (score)	12.09±2.964	12.58±3.414		2146.0	0.350
Step (score)	76.80±22.295	74.41±18.649		2224.0	0.750
BBS (score)	54.76±1.875	54.60±6.092		2083.5	0.090
PFAQ (score)	1.36±3.988	0.38±0.732		2209.5	0.250

Abbreviations: PwCD, possible cognitive decline; ꭓ^2^, chi-square; U/T, independent T; p, significance; ±, standard deviation; MMSE, Mini-Mental State Examination; MoCA, Montreal Cognitive Assessment; CDT, Clock Drawing Test; VF, Verbal Fluency Test; STS, Sit-to-Stand Test; Step, Step Test; BBS, Berg Balance Score; PFAQ, Pfeffer Functional Activities Questionnaire.

Source: Prepared by the authors.

As expected, significant differences were observed across all cognitive measures, with the PwCD group showing poorer performance on the MMSE, MoCA, CDT, and VF instruments (Table 1). Regarding physical capacities, no significant between-group differences were observed in lower limb strength, cardiovascular endurance, or balance, as assessed by the STS, Step Test, and BBS, respectively (Table 1).

**Figure 1:**
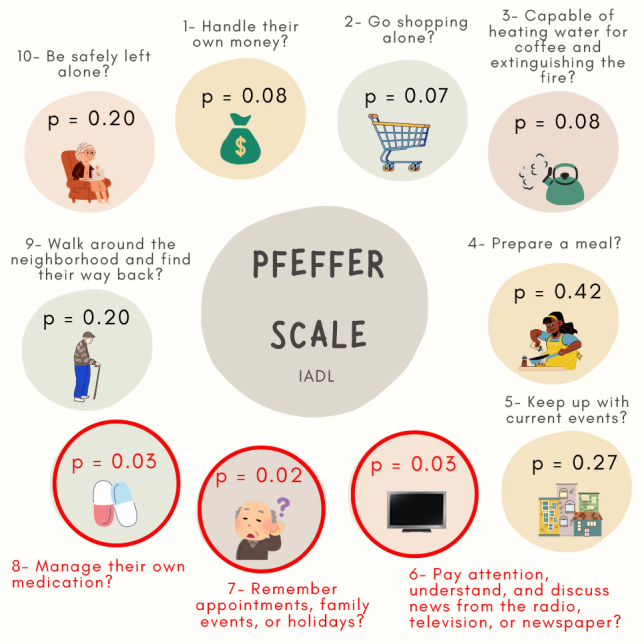
Presentation of the ten domains of instrumental activities of daily living (IADLs) assessed by the Pfeffer scale. A significant difference was observed in IADLs domains 6, 7, and 8 between healthy older adults and those with possible cognitive decline. The remaining domains did not demonstrate significant differences.

Source: Prepared by the authors using Canva.using Canva.

With respect to functionality, no significant difference was observed in total Pfeffer scale scores between groups (Table 1). However, based on the IADLs cutoff point, older adults with possible cognitive decline showed a higher prevalence of functional impairment (Healthy=12.5%; PwCD=87.5%). Item-level analysis revealed greater impairment in the PwCD group in tasks related to attention capacity (Healthy=0.0%; PwCD=7.2%), recall of appointments (Healthy=21.5%; PwCD=25.5%), and medication management (Healthy=4.6%; PwCD=7.2%). Figure 1 presents between-group differences for each IADL assessed by the Pfeffer scale.

**Figure 2:**
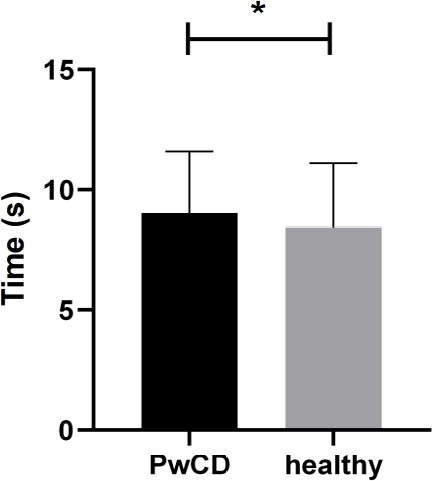
Dual-task naming animals test, time variable (seconds), for the groups with possible cognitive decline (PwCD) and healthy controls.

Notes: Mann-Whitney U test for independent samples. *p=0.04.

Source: Prepared by the authors.

**Figure 3:**
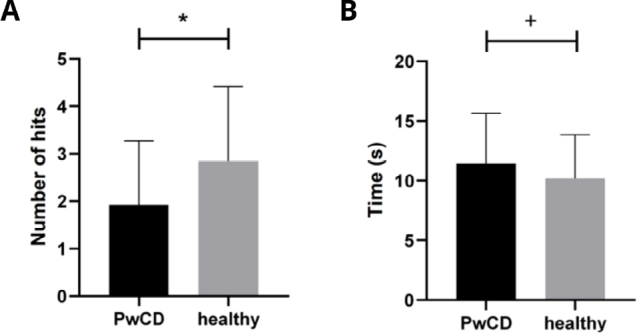
Performance on the dual-task-serial 7s test for groups with possible cognitive decline (PwCD) and healthy controls. (A) Number of correct responses – higher values indicate better performance. (B) Response time (seconds) – lower values indicate better performance.

Notes: Mann-Whitney U test for independent samples.

In analyses examining the association between DT performance and functionality, PwCD participants showed poorer performance on the TUGdt-NA time (Figure 2) and TUGdt-S7 performance, both for the number of correct responses (Figure 3A) and time (Figure 3B), despite no differences in simple motor task performance as assessed by the STS test. Table 2 summarizes the comparative analyses of functionality, DT performance, and DTC between groups.

*p<0.001; +p=0.027.

Source: Prepared by the authors.

**Table 2:** Comparative analysis of the variables of functionality, gait, dual-task performance, and dual-task costs among the groups.

	PwCD (n=55)	Healthy (n=89)	U/T	p-value
TUG (sec)	7.386±1.5907	6.781±1.3743	2031.0	0.120
TUGdt-NA (sec)	11.055±3.9580	8.505±2.4980	1918.0	0.040
TUGdt-NA (score)	5.88±2.100	6.32±1.705	1990.0	0.080
DTC NA	28.863±24.267	25.325±28.231	2170.0	0.250
TUGdt-S7 (hits)	1.63±1.188	2.53±1.559	1563.0	<0.001
TUGdt-S7 (sec)	13.565±3.7471	10.465±3.8389	1898.5	0.027
DTC S7	61.875±40.429	52.297±46.259	2008.0	0.070

Abbreviations: PwCD, possible cognitive decline; U/T, independent T; p, significance; TUG, Timed Up and Go test; TUGdt-NA, Timed Up and Go test with dual-task naming animals; TUGdt-S7, Timed Up and Go test with dual-task serial 7s; DTC NA, dual-task cost naming animals; DTC S7, dual-task cost serial 7s.

Source: Prepared by the authors.

Regression analyses showed that poorer performance times on the TUGdt-NA and TUGdt-S7 were associated with a 21% and 14% lower likelihood of preserved functionality, respectively (TUGdt-NA: odds ratio [OR] 0.79; 95% confidence interval [CI] 0.66–0.96; p=0.01; TUGdt-S7: OR 0.86; 95%CI 0.75–0.99; p=0.04). These associations were no longer significant after adjustment for education, race, and global cognition (TUGdt-NA: OR 0.81; 95%CI 0.50–1.31; p=0.40; TUGdt-S7: OR 0.92; 95%CI 0.76–1.13; p=0.45). In item-specific analyses, poorer performance on the TUGdt-S7 was associated with a 35% reduction in the likelihood of being able to prepare a meal (OR 0.65; 95%CI 0.44–0.95; p=0.02), whereas no significant associations were observed for the remaining items. No significant associations were found for any Pfeffer item in the TUGdt-NA.

## DISCUSSION

The present study examined whether DT and functional assessment tools can help detect cognitive impairment in older adults without a prior diagnosis. Although no differences were observed between groups in age, sex, comorbidities, STS, Step Test, BBS, or total Pfeffer scores, the PwCD group showed poorer performance in IADL, particularly in tasks related to attention, recall of appointments, and medication management, as well as worse DT performance. These findings suggest that DT performance may reflect functional capacity and represent a promising tool for the early detection of cognitive decline in older adults.

Older adults with PwCD may experience increasing difficulty in activities that require more complex cognitive processing, such as rapid decision-making, planning precise movements, and coordinating multiple tasks simultaneously^16^. Understanding these differences is essential for developing intervention strategies and preserving functionality, particularly among individuals at risk of neurodegenerative diseases. As expected, PwCD participants showed preserved functionality in basic ADLs, consistent with their independent profile and engagement in regular physical and social activities. Despite the absence of differences in total Pfeffer scores, the application of a specific cutoff revealed a higher prevalence of functional impairment in the PwCD group. Therefore, the analysis focused on individual items rather than the total score, revealing greater difficulty in specific IADLs that may represent early indicators of cognitive decline. These findings suggest that the Pfeffer scale may have limited sensitivity for distinguishing healthy older adults from those with early-stage PwCD, highlighting the importance of detailed, item-level functional assessment.

When more complex IADLs were considered, greater impairment was observed in the PwCD group, likely due to higher motor and executive function demands. Lower performance was evident in tasks related to attention, memory for appointments, and medication management. Impaired attention reflects difficulty maintaining focus and concentration, which may hinder the efficient execution of daily tasks and increase difficulty in simultaneous activities. A decline in remembering appointments may compromise autonomy, time management, and personal organization. Functionally, individuals capable of performing multiple tasks simultaneously tend to manage their time more efficiently than those performing tasks sequentially^28^. Difficulties with medication management also reflect impairments in attention and working memory. Thus, DT performance is relevant to many daily activities, and increased task complexity is expected to exacerbate functional difficulties.

In the TUGdt-NA and TUGdt-S7 tests, older adults with PwCD showed poorer performance, indicating a lower likelihood of maintaining preserved functionality. Poorer performance on the TUGdt-NA was associated with a 21% reduction in the likelihood of preserved functionality. The animal naming task requires working memory and semantic retrieval, cognitive domains that are frequently impaired in individuals with PwCD, who often experience difficulties in retaining short-term information and performing complex tasks^29^. These cognitive demands may partly explain the poorer performance observed under this condition. Similarly, poorer performance on the TUGdt-S7 was associated with a 14% reduction in preserved functionality. Serial subtraction tasks require sustained attention, concentration, and logical reasoning, and performance may be influenced by educational level, which can act either as a facilitator or a barrier to task execution. In Brazil, persistent challenges in mathematics education, reflected in national and international assessments, may further increase the difficulty of this task for individuals with lower educational attainment^30^. In contrast, animal naming relies more heavily on semantic memory and the retrieval of previously stored information^31^. Thus, different DT conditions engage distinct cognitive resources, and these differences tend to become more pronounced in the presence of cognitive decline.

Complementing the analyses based on the total Pfeffer score, item-specific logistic regression analyses were conducted to examine functional performance in greater detail. Among the functional items, poorer performance on the TUGdt-S7 was associated with a 35% reduction in the likelihood of being able to prepare a meal, whereas no significant associations were observed for the remaining items. No significant associations were found for any Pfeffer item in the TUGdt-NA. Being able to prepare a meal is an IADL that relies on attention, planning, working memory, and cognitive flexibility, which may explain its more consistent association with DT performance^16^. However, these findings should be interpreted with caution, as the sample consisted of robust, community-dwelling older adults who participated in structured physical activity programs, with functional independence as a participation criterion. Consequently, functional dependence was rare across most Pfeffer items, and the lack of significant associations likely reflects the limited functional variability of the sample rather than the absence of a relationship between DT performance and functional status.

DT gait tests, such as the TUGdt-NA and TUGdt-S7, have been recommended for predicting future dementia incidence and estimating the time to conversion from mild cognitive impairment to dementia^15,32^. Unlike functional scales and questionnaires based on subjective observation of daily activities, these tests provide objective measures with adequate specificity and sensitivity^33^. They also allow the assessment of variables such as test completion time and DTC, with established cutoff points associated with dementia progression^15,32,34^. Moreover, these instruments are low-cost, easy to administer, and feasible even in settings with limited human resources and infrastructure.

This study has some limitations that should be acknowledged. Its cross-sectional design precludes causal inferences and limits conclusions regarding the temporal direction of the observed associations. In addition, the sample consisted of physically active, community-dwelling older adults with healthy lifestyle behaviors, which may restrict the generalizability of the findings to the older population as a whole.

In conclusion, DT performance appears to be closely associated with functional capacity in older adults. These findings contribute to a better understanding of the relationship between cognition and functionality and underscore the relevance of incorporating objective DT assessments into functional evaluation. DT gait tests may represent a practical and low-cost tool for the early identification of functional dependence, supporting timely interventions, preventive strategies, and public policies aimed at preserving independence and quality of life in aging populations.

## Data Availability

The datasets generated and/or analyzed during the current study are not publicly available due to ethical and privacy restrictions but are available from the corresponding author upon reasonable request.
